# Evaluation of the effect of mental health training of primary health care workers on attitudes towards mental illness

**DOI:** 10.1192/j.eurpsy.2021.1055

**Published:** 2021-08-13

**Authors:** O. Buhari, A.J. Ogunmodede, O. Adegunloye

**Affiliations:** 1 Dept Of Behavioural Sciences, UNIVERSITY OF ILORIN & University OF ILORIN TEACHING HOSPITAL, ILORIN, Nigeria; 2 Dept Of Behavioural Sciences, UNIVERSITY OF ILORIN TEACHING HOSPITAL, Ilorin, Nigeria

**Keywords:** Primary health care workers (PHCWs), attitude, Mental health training, Mental illness

## Abstract

**Introduction:**

The World Health Organization (WHO) set a target of task shifting as a means of achieving improved mental health services within the community as a means of tackling the unmet needs of mental health care. Primary health care workers (PHCWs) have been identified as essential to achieving this goal.

**Objectives:**

This study was to identify attitudes and beliefs of PHCWs on mental illness, and to assess the effect of a mental health training on these attitudes and beliefs.

**Methods:**

The attitude towards mental illness (ASMI) scale was administered on 91 PHCWs pre- and post- a 4 day training on mental health to assess change in attitude across 6 domains.

**Results:**

Our findings revealed significant positive change in four domains, namely separatism (p = < 0.001), restrictiveness (p = < 0.001), benevolence (p = p =< 0.001) and stigmatization (p = < 0.001). The changes in stereotyping (p = 0.475) and pessimistic prediction (p = 0.056) domains were not clinically significant.

**Conclusions:**

Primary health care workers’ negative attitude and stigmatizing beliefs can be improved upon via regular enlightenment programmes and training. This can be done at regular intervals

